# Nutrition and Neuroinflammation: Are Middle-Aged Women in the Red Zone?

**DOI:** 10.3390/nu17101607

**Published:** 2025-05-08

**Authors:** Veronique Bernier, Angeline Chatelan, Camille Point, Mélanie Strauss

**Affiliations:** 1 Department of Psychiatry, Brugmann University Hospital, Université Libre de Bruxelles—ULB, 1020 Brussels, Belgium; 2 Department of Nutrition and Dietetics, Geneva School of Health Sciences, HES-SO University of Applied Sciences and Arts Western Switzerland, CH-1227 Geneva, Switzerland; 3 Department of Neurology and Sleep Unit, Université Libre de Bruxelles—ULB, Hôpital Universitaire de Bruxelles (H.U.B), CUB Hôpital Erasme, Route de Lennik 808, 1070 Bruxelles, Belgium; 4 Laboratory of Experimental Neurology, Université Libre de Bruxelles—ULB, Route de Lennik 808, 1070 Bruxelles, Belgium

**Keywords:** menopause, inflammation, nutrition, depression, Alzheimer’s disease, sleep, estrogen, overweight

## Abstract

Women exhibit unique vulnerabilities in health, especially regarding mental health and neurodegenerative diseases. Biological, hormonal, and metabolic differences contribute to sex-specific risks that remain underrepresented in clinical studies. Diseases such as major depressive disorder (MDD) and Alzheimer’s disease (AD) are more prevalent in women and may be influenced by hormonal transitions, particularly during menopause. Chronic low-grade inflammation is emerging as a shared mechanism underlying both conditions, and this inflammatory state can be worsened by dietary habits. During menopause, mood and sleep disturbances can influence dietary behavior, leading to enhanced snacking and consumption of high-glycemic and comfort foods. Such foods, low in nutritional value, promote weight gain and elevated inflammatory markers. Their consumption combined (or not) with a preexisting Western diet pattern—already linked to inflammation—could reinforce systemic inflammation involving the gut–brain axis. Moreover, the symptoms “per se” could act on inflammation as well. Peripheral inflammation may cross the blood–brain barrier, sustaining mood disorders and promoting neurodegenerative changes. Finally, MDD and AD are both associated with conditions such as obesity and diabetes, which occur more frequently in women. The review highlights how menopause-related changes in mood, sleep, and diet may heighten susceptibility to mental and neurodegenerative diseases.

## 1. Introduction

Women represent a uniquely vulnerable population in health research, particularly in relation to mental health and neurodegenerative disorders. Thus, approximately 185 million people worldwide suffer from major depressive disorder (MDD) with a higher risk (RR: 1.602 (1.619–1.620)) and prevalence in females (170.4 per million) compared to males (109.2 per million) [1]. MDD as defined by the Diagnosis Statistic Manual of Disorders (DSM-5) criteria has a multifactorial etiology, influenced by socio-economic, environmental, and biological factors. Since 2015, MDD has ranked as the leading cause of disease burden among women [2]. Likewise, women account for nearly two-thirds of all Alzheimer’s disease (AD) cases [3], and at age 45, their lifetime risk of developing AD is approximately double that of men (20% vs. 10%) [4]. AD, the most common cause of dementia, currently affects an estimated 58 million individuals worldwide—a number projected to rise to over 150 million by the 2050s [5]. AD is widely acknowledged as a multifactorial disease, with complex interactions between genetic predispositions such as APOE-ε4 status [6] and environmental risk factors. These include diet, cardiovascular and metabolic conditions, psychological stressors, and sleep disturbances, all of which are rising in prevalence globally [7]. The increased vulnerability of women has traditionally been attributed to greater longevity. However, growing evidence points to additional contributing factors, including genetic influences (particularly APOE-ε4), socio-cultural determinants such as education and occupational status, and also hormonal transitions such as menopause and reproductive history [8,9]. MDD and Alzheimer’s disease (AD) share overlapping symptoms such as depressive mood, anxiety, cognitive decline, and sleep disruption, and they are both associated with low-grade chronic inflammation [10,11,12]. Furthermore, MDD has been identified as a potential risk factor for the later development of AD [13,14]. The shared characteristics and gender-specific aspects of both conditions have led us to investigate a particular hormonal phase in women: menopause.

Beyond the well-recognized risks such as cardiometabolic disease and bone fragility, menopause is increasingly understood as a window of heightened vulnerability to mood disorders, sleep disturbances, and cognitive disorders. The menopausal transition—defined by a progressive decline in estrogen and progesterone—typically begins with perimenopause (around age 45) and culminates in menopause, clinically defined as 12 consecutive months of amenorrhea, occurring on average between ages 50 and 52 [15,16]. This hormonal transition is linked to an increased risk of mood disturbances, including depression (36.6% during perimenopause, 30.2% during postmenopause), anxiety (55.7% and 52.5%), and sleep [17] and cognitive disorders [17,18]. Notably, perimenopause, due to the fluctuation of hormone levels, appears to be an even more vulnerable period than menopause itself. In addition to mood and sleep disturbances, the menopausal transition is associated with various physiological changes, including metabolism slowdown and elevated inflammatory markers [19].

Nutrition emerges as a critical factor in this context, both as a modifiable risk and as a mediator of inflammation and symptom expression. The Western diet (WD)—one of the most dominant worldwide diet patterns and characterized by high intakes of saturated and trans fats, elevated ratio omega-6/3, refined carbohydrates, sugars, salt, and ultra-processed foods—is strongly associated with elevated inflammatory markers such as C-reactive protein (CRP) [20,21]. Simultaneously, the WD is deficient in anti-inflammatory nutrients, including dietary fiber, omega-9 fatty acids, and antioxidants. Importantly, symptoms like stress, low mood, and poor sleep, highly frequent in menopause, may drive preferences for sweet-tasting and comfort foods rich in sugar and fat, exacerbating the inflammatory potential of the WD. Moreover, metabolic changes could enhance weight gain that could also act in this context.

This review focuses on the specific vulnerability of women to neurodegenerative and mental diseases, highlighting menopause and nutrition as potential critical mediators. We point out the symptoms and physiological changes during the menopausal period that could impact nutrition. Then, we outline the underlying mechanism of a pro-inflammatory diet and the effects of mood and sleep disturbances “per se” on inflammation. Finally, we conclude by explaining why a chronic inflammatory state could be a risk factor for these pathologies.

## 2. Consequences of Hormonal Changes on Mood and Sleep Disorders, Metabolism, and Nutrition

### 2.1. Mood and Cognitive Disorders

The underlying mechanism that links physiological change to mood issues could rely on estrogen hormonal fluctuations [22,23]. Women have two higher periods of vulnerability toward depression than men which are the prepubertal age (young women) and the perimenopausal/menopausal period (middle-aged women) [2,24]. Perimenopause has the highest range of hormonal fluctuations consistent with a higher level of mood disorders than menopause, as aforementioned [23,25]. 

In the brain, the hippocampus and hypothalamus are thought to be involved, as they contain estrogen receptors—with a particularly high concentration in the hippocampus [26]. The hippocampus is engaged in emotional and cognitive functions, such as memory and controlled attention, and both functional and structural alterations have been observed in depression [27,28]. Its volume has also been shown to vary with the level of estrogen [27]. In addition, hippocampal plasticity could fluctuate due to endogenous or environmental factors such as aging, nutrition, and anxiety [29,30,31,32,33]. Likewise, the amygdala also contains estrogen receptors, and its hyperactivity has been associated with depression [34]. The administration of estrogen into this specific brain region has been demonstrated to reduce anxiety [34]. However, further research that considers the neuroanatomical complexity of the amygdala is necessary to better understand its role in depression, particularly in an inflammatory context [35,36].

Steroid hormones like estrogen could have neuromodulator effects impacting γ-Aminobutyric acid (GABA) and serotonin levels [22,37]. Estrogen is associated with an increased activity, sensitivity, and receptor expression of endogenous opioids such as beta-endorphin [38]. The plasma level of beta-endorphin in menopausal women has been shown to be significantly lower [39]. Beta-endorphin can modulate the GABA receptor and lead to the accumulation of dopamine in the brain reward system, and it could be involved in homeostasis-restoring behaviors [40]. Ovariectomy and menopause are associated with lower concentrations of plasma beta-endorphins in women and in contrast, estradiol therapy could reverse this effect [39,41,42]. A decrease in estrogen has been linked to depression and anxiety [43]. In contrast, hormone-replacement therapy during perimenopause may help to prevent postmenopausal depression in women [44]. Similarly, the use of oral contraceptives has been shown to moderate symptoms of depression and anxiety compared to non-use [45].

The decline in estrogen is associated with cognitive impairments which are sub-symptoms or core symptoms of menopause, depression and AD [46]. About two-thirds of women have cognitive complaints during perimenopause, despite objective cognitive tests potentially failing to detect these changes [18]. A lifelong endogenous exposition of estrogen as well as hormonal therapy in elderly women have been shown to be protective of cognitive status [47,48]. Overall, these results suggest that estrogen deprivation could play a role in the etiology of mood and cognitive disorders and that hormone therapy may be protective during pre- and postmenopause, as female brain plasticity remains sensitive to estrogen over time [47,49].

### 2.2. Sleep Disturbance

The incidence of sleep disturbance ranges from 16% to 47% during perimenopause and 35% to 60% during postmenopause in women [17]. Women in these stages experience poorer sleep quality, characterized by increased sleep latency and greater sleep fragmentation. Hormonal changes have direct consequences on vigilance systems. Estrogen promotes sleep by modulating norepinephrine, serotonin, and acetylcholine metabolism, and progesterone exerts sedative and anxiolytic effects by stimulating benzodiazepine and GABA receptors, helping to maintain deep sleep [17,50]. Decreased melatonin levels are also associated with reduced subjective sleep quality [51]. In addition to direct hormonal effects, vasomotor symptoms, such as nighttime hot flashes, disrupt sleep cycles, leading to frequent awakenings and reducing sleep quality [52]. Comorbidities impacting sleep are also more frequent in postmenopausal women. They are at higher risk of mood disturbances, obstructive sleep apnea—due to weight gain and loss of protective estrogen effects—and restless leg syndrome, leading to increased insomnia disorders and sleep fragmentation [24,53,54].

Objective polysomnographic data on sleep changes in menopausal women are scarce, but the available studies tend to confirm longer sleep latencies, reduced total sleep time, and lower sleep efficiency [55,56,57], while age may be a significant contributing factor in these observations [57].

### 2.3. Mood Disorder, Sleep Disturbance, and Nutrition

Craving for snacks and sweets when feeling anxious or depressed is a common feature [58,59,60,61,62,63]. Likewise, neurocognitive changes occur due to poor sleep, particularly in the prefrontal cortex and reward centers, making individuals more likely to seek out immediate energy-dense comfort foods [64,65,66]. Thus, sleep disturbance, poor sleep, or lack of sleep significantly increase food intake, especially high-glycemic foods (HGI). HGI foods have the ability to rapidly increase glycemia (in a physiological range) and trigger an insulinemia spike, fostering glucose storage in cells and leading to weight gain [20,67,68]. Sweet-tasting food, soft drinks, and comfort food have a high glycemic index (HGI). HGI foods are rich in sugar and/or refined carbohydrates (CHOs) [20]. Sugars refer to monosaccharides (mainly glucose and fructose) and disaccharides (sucrose). Sucrose is the white or brown sugar commonly used and is also composed of glucose and fructose. Refined carbohydrates (CHOs) come mainly from grains (e.g., wheat, rice) or potatoes and are constituted of long chains of glucose (starch). CHOs are ultimately catabolized in glucose by enzymatic reaction in the intestinal tract. Due to the highly processed grains, high-temperature cooking processes, and low fiber content that define refined carbohydrates (e.g., white bread, cornflakes, chips), starch becomes more accessible to the enzymatic process than whole grains. Therefore, the speed of glucose release is significantly higher than in non-refined carbohydrates, as is glycemia. 

Comfort food also refers to highly palatable food [69]. Palatability is a complex definition that relies on an ingestor, an ingestant, a response measure, and an observer [70]. Briefly, a highly palatable food compared to a regular one leads to an increase in the amount ingested [70]. Smooth and highly processed textures, high sensorial properties, and fat and sugar content increase palatability. Ultra-processed foods (UPFs) are often high-energy-density and highly palatable foods and therefore comfort foods [69]. Palatability has been associated with the release of endorphins, increasing reward and pleasure [71]. Likewise, evidence has shown that glucose has the ability to trigger the release of dopamine along with endogenous opioids (endorphin) in the brain and, in particular, through the reward system [72,73,74]. Therefore, it could explain why the consumption of sugar and comfort food could contribute to enhancing mood, becoming a form of self-medication. Moreover, as we wrote above, the menopause period “per se” is associated with an decrease in the opioid level that could perhaps contribute to fostering the “call” for glucose as well. Intermittent and frequent intake of glucose such as snacking could also lead to another issue. Evidence shows in model animals that neural adaptation could occur in such conditions and lead to a “drug-like effect” commonly called sugar addiction [72,73,74]. This result could also enhance the increase in the dietary intake of comfort food and sugar [72,73,74]. Consistently, high-glycemic food has been associated with mood disturbances and depression in adults and postmenopausal women [67,75,76].

### 2.4. Impact of Decreasing Estrogen Levels on Metabolism and Weight

The deficiency of estrogen occurring in menopause has a direct impact on metabolism and physiology, affecting lipid metabolism and energy consumption and fostering the storage of fat mass in abdominal/visceral parts of the body [77,78,79]. It has been well demonstrated that menopause is associated with weight gain. For instance, middle-aged women gained 0.7 kg per year in the SWAN study, whereas in the Nurse Health Study they gained an average of 3 kg over 8 years of follow-up, for an average of 0.4 kg [77,80]. Overall, the lean mass (muscles) decreases, whereas the fat mass increases [79]. As a result, menopause and perimenopause are associated with an increase in abdominal fat mass. In contrast, hormonal therapy (estrogen) could partially modulate the increase in abdominal fat and obesity [77,79]. Fat mass, in particular abdominal fat, could act as an endocrine organ and trigger the release of pro-inflammatory cytokines as well as fostering cardiometabolic comorbidities [77]. While menopause “per se” is not the only risk factor, obesity is more prevalent in women in the middle-age and older age group than men [78,79]. Interestingly, obesity is also associated with a higher scale of severe menopausal symptoms assessed by the Blatt-Kupperman menopausal index or Menopause-specific quality of life questionnaire (MENQOL). Both questionnaires contain mood and sleep disturbance and cognitive impairment criteria [81,82].

In addition, body weight dissatisfaction is common among middle-aged women. In a previous study, we showed that 13% and 33% of women aged 50–64 years from the general Swiss population were very dissatisfied or dissatisfied with their body weight, respectively, and 56% of them wanted to lose weight [83]. Weight gain and abdominal fat accumulation during the perimenopause could, in part, result from alterations in dietary factors, such as changes in food and nutrient intake as well as dietary behaviors. Such dietary changes may be driven by reduced endogenous opioid production, mood disturbances, and sleep disturbance as aforementioned. In this context, dietary preferences may shift toward comfort food [84,85,86]. On the other hand, some women may proactively improve their diet quality during the menopausal transition in response to physiological alterations, anticipated health concerns, or body dissatisfaction.

Research assessing whether specific dietary factors change concomitantly with the menopausal transition remains limited. The few available studies on this topic have reported either a slight decline in total energy and macronutrient intake or no changes [87,88,89,90]. For instance, the MONET study followed a small convenient sample of non-obese Canadian women who completed a 7-day food diary every year for 5 years. It revealed a reduced intake of total energy (kcal/d), protein (g/d), fat (g/d), and dietary fiber (g/d) and an increased number of eating occasions (more frequent intake or snacking) over time. However, these changes were rather related to time (becoming older) than changes in menopausal status [89]. Similarly, in Switzerland, Grisotto and colleagues, who followed middle-aged women of the general population for 5 years, found that dietary intake had not changed much before or after menopause [91]. In comparison to women who remained in the premenopausal state (*n* = 259), the intake of pastries and added sugar was even slightly reduced the post-menopause (−1.8 g/d (−5.4, 1.8) *p* = 0.34; −0.9 g/d, 95% CI: −2.0, 0.2, *p* = 0.11, respectively, *n* = 229). However, this cohort study might have missed short-term dietary changes because food-frequency questionnaires were completed only twice within 5 years. Finally, MacDonald et al. reported a reduction in the consumption of red and processed meat, bread, biscuits, and cakes, alongside an increase in the intake of poultry, non-oily fish, cereals, rice/pasta, and fruit, suggesting a shift toward a healthier dietary pattern following the menopausal transition [87]. However, the study was limited by the analysis of only two dietary assessment points over a five-year period and the absence of a comparison group consisting of women who remained premenopausal. Of note, none of these studies stratified women by important risk factors (e.g., depressed mood, disturbed sleep, or body weight dissatisfaction).

In sum, the existing data show that most women gain weight during menopausal transition and a large proportion of middle-aged women are dissatisfied with their body weight. The few aforementioned cohort studies investigating whether women change their dietary intake during the menopausal transition have shown slight or no changes. Thus, we could hypothesize on this basis that a WD context, associated with metabolism slowdown and potential increased snacking in the menopausal transition, could produce a gradual weight increase [89]. However, when taking our context into consideration, the reported epidemiological studies suffer from a lack of robust methodological designs, as previously mentioned, and adjusted dietary assessment methods, e.g., risk of underreporting of comfort food due to social disability bias (higher in women dissatisfied with body weight) or inability to detect small changes with food-frequency questionnaires, especially in behaviors (e.g., snacking) or consumed quantities. Therefore, small and specific changes and changes in behaviors may remain undetected.

Overall, the data are consistent with the hypothesis that it is more likely small dietary changes occurring for several years of perimenopause that are responsible for weight gain rather than drastic changes in dietary intake. Thus, a small gap between daily energy intake and expenditure (e.g., 100 kcal/d) or small changes towards foods with a high glycemic load (GL, calculated by multiplying the carbohydrate quantity of food by the glycemic index and dividing by one hundred) could be sufficient to gain weight and would be hardly detected in a food-frequency questionnaire or other dietary assessment methods used by dietitians [92,93]. Therefore, well-designed large-scale cohort studies to better understand small dietary changes at the food group level (not just at the nutrient level) and small changes in GL, especially among women with higher risk factors, are needed. This could lead to more targeted healthy eating strategies to limit the adverse biological outcomes of perimenopause.

## 3. Etiology of a Pro-Inflammatory Diet

### 3.1. Dietary Intake and the Activation of Pro-Inflammatory Immune Pathways

**Sugar**. A high-sugar diet has been widely associated with systemic inflammation which could be the result of an immune system impairment and/or an alteration of the gut permeability [94,95,96,97]. Sugar refers mainly to glucose, fructose, and sucrose, as aforementioned. While fructose and glucose have the same formula and are isomers, they have different metabolization pathways. A key difference between them is the lack of control or feedback loop towards fructose dietary intake compared to glucose. In the gut, glucose absorption has an energy-requiring process mediated by a sodium co-transporter, while fructose is mainly absorbed by enterocytes through facilitated passive transport. Glucose can be metabolized by each cell of the body, while fructose is mainly metabolized in the liver [98]. Furthermore, the metabolization of fructose does not require insulin; thereby, no regulation process can control an excessive supply of fructose in hepatocytes [99]. Dietary sugars and fructose, in particular, have the ability to promote the de novo synthesis of free fatty acids (DNL) [98]. Therefore, an excess and unregulated fructose load could lead to an accumulation of free fatty acid precursors that could promote DNL but also lead to metabolite byproducts such as lactate or uric acid, which could in turn produce oxidative stress and inflammation [98,100,101,102,103,104].

Fructose could also act through the pro-inflammatory role of the advanced glycation end products (AGEs). AGEs are the result of a non-enzymatic reaction between a reduced sugar and a free amino acid and lead (mainly) to protein glycation. They are normal byproducts of metabolism, but their accumulation is associated with oxidative stress and inflammation [105,106]. AGEs could also come from exogenous sources such as food [105,106]. An excessive intake of dietary fructose has been shown to be more associated with protein glycation, oxidative stress, and pro-inflammatory cytokines in peripheral mononuclear blood cells than glucose [105,107]. Likewise, in human dendritic cells, Jaiswall at al. showed in vitro that high-fructose compared to high-glucose conditions resulted in a greater increase in the level of intracellular AGEs. Furthermore, this study showed that AGEs through the activation of their specific receptor RAGE (receptor of advanced glycation end products), leads to the activation of the transcription nuclear factor kappa-B (NF-κB) pathway and the release of pro-inflammatory cytokines [105,108]. Thus, a high-fructose diet intake increases the AGE level, leading to the activation of RAGE and subsequently inflammation. Finally, inflammation has been shown to upregulate the expression of RAGE, thus constituting an inflammatory vicious circle [105].

While glucose is essential for a proper immune function, high glucose (HG) exposure has also been shown to have a deleterious effect on immune cells [95,104]. HG could upregulate the expression of Toll-like receptors (TLRs), increase their reactivity and in turn lead to triggering the NF-kB pathway and the release of pro-inflammatory cytokines [104,109]. Likewise, Xu et al. showed that macrophages exposed to high amounts of glucose could shift to a pro-inflammatory phenotype through the activation of the transforming growth factor-β-activated kinase 1 (TAK1) and lead to the release of pro-inflammatory cytokines [110]. Despite different metabolic pathways as aforementioned, fructose and glucose seem to be equally involved in inflammatory issues and no prominent difference has been shown between them [96]. Interestingly, added sugars that come from beverages (e.g., soft drinks, tea, coffee) independently of their nature have been associated with higher inflammatory markers and elevated glycemia than solid sugars in food [111].

Consistently, refined CHOs with a high glycemic index are also associated with oxidative stress, activation of the pro-inflammatory NFkB pathway and elevated inflammatory markers (CRP) [20,94,112,113]. In contrast, low-glycemic index CHOs are associated with a decrease in the CRP level [114].

**Fat.** Dietary fats are presumed to have pro-inflammatory effects as well. Saturated fatty acids (SFAs) have been shown to be associated with inflammation through the activation of TLR-4, the production of ceramide, and the formation of lipid rafts in the cell membrane [115]. A high-fat diet is associated with an elevated uptake of LPS in the gut. LPS can cross the intestinal barrier via transcellular pathways such as the chylomicrons (lipid absorption) or paracellular routes such as a leaky gut [116]. SFA can trigger TLR-4 into the condition of a previous activation of the receptor (e.g., LPS or oxidative stress) [117]. A high-fat diet can lead to the intracellular accumulation of palmitic acid. This can enhance the production of reactive oxygen species (ROS) and the release of pro-inflammatory proteins [118]. Likewise, the trans fatty acids which come mainly from industrially hydrogenated fat and fried foods can activate the pro-inflammatory pathway NFκB and are associated with an increase in the CRP level [119,120]. Furthermore, in scavenger cells, it has been shown that when the cellular capacity to metabolize oxidized cholesterol is exceeded, this leads to the intracellular accumulation and molecular crystallization of CT which can damage the cell, thus activating pro-inflammatory pathways and releasing pro-inflammatory cytokines [121]. Finally, the n-6 polyunsaturated fatty acids (AGPIs), the so-called omega-6, are precursors of the prostaglandin and leukotriene proteins which are essential for the onset of inflammation. On the contrary, the n-3 AGPIs, omega-3, have anti-inflammatory properties and are the precursors of maresine, protectine, and resolvine which are the mediators of the final state of inflammation. Both precursors n-6 and n-3 use the same enzymatic pool to synthetize their metabolites. Therefore, the ratio between both is important to consider. Still, the WD has an elevated n-6/n-3 ratio that can contribute to enhancing an inflammatory context [122,123,124].

### 3.2. Diet and Gut Permeability

Gut integrity is a major issue for our immune system. In particular, the thickness of the gut mucus, which constitutes a physical barrier protecting the enterocytes as well as the tight junction’s expression, are critical points for cellular barrier integrity. When the gut barrier is damaged, the so-called “leaky gut” could lead to the translocation of endotoxins or Pathogen Associated Molecular Patterns (PAMPs) such as LPS into the subepithelial tissue and the bloodstream, subsequently leading to local and systemic inflammation [125,126]. Endotoxemia or metabolic endotoxemia was first defined by Cani et al. as an increase in the plasma LPS level induced by a high-fat diet and associated with low-grade inflammation [126,127]. It has been shown that PAMPs can bind the Toll-like receptors (TLR2 and 4), leading to the release of pro-inflammatory cytokines that could in turn foster systemic low-grade inflammation [127,128,129]. A high-fructose, high-sucrose diet could induce oxidative stress in the endoplasmic reticulum of the enterocyte, leading to downregulated expression of the tight junction and thereby an increase in gut permeability [103]. Moreover, fructose could impair the homeostasis of nitric oxide that could also act in this context [130].

The gut microbiota is defined by the microorganisms (mainly bacteria) which are hosted and live symbiotically in our intestinal tract [131]. The microbiota diversity and abundance are protective for our health and immunity as certain bacteria species can enhance (among other health benefits) the thickness of the intestinal mucus and promote the upregulation of tight junctions and the release of metabolic byproducts such as short fatty acids (SCFAs)—acetate, propionate, and butyrate. SCFAs have a trophic role for enterocytes (butyrate), enhance the intestinal barrier function (butyrate) by fostering the expression of tight junctions, and have anti-inflammatory properties [132,133,134]. The microbiota composition is presumed to change during the aging process. Dysbiosis, which refers to both quantitative and qualitative disruptions in microbiota composition, has been shown in elderly individuals [135,136]. Additionally, aging is linked to chronic inflammation, or “inflammaging”, which is considered as a risk factor for various diseases [137]. Notably, a study on animal models has shown that transplanting the microbiota of aged mice into young animals can induce “age-related chronic inflammation”. This result may indicate a contribution of the gut microbiota to the etiology of inflammaging [136]. The microbiota composition is also highly impacted by dietary intake. A high-fructose diet could lead to dysbiosis [138,139]. Thus, a high intake of fructose could cause a reduction in butyrate-producing bacteria and promote Gram-negative bacteria and LPS in the gut. LPS could also trigger macrophage activation and the release of pro-inflammatory cytokines which can affect the tight junction and consequently weaken the intestinal permeability and enhance inflammation [130,139,140]. Likewise, a high sugar dietary intake has been shown to induce a shift of composition from Firmicutes and SCFA-producing bacteria to Gram-negative bacteroidetes and proteobacteria species. This dysbiosis has been shown to promote intestinal permeability, endotoxin translocation, and inflammation, as aforementioned for fructose only [141,142].

Dietary fiber (DF) derived from plant-based foods is the main substrate of the gut microbiota and is presumed to shape the diversity and abundance of the bacterial species within it [143]. Although the impact of DF on bacterial diversity, as well as the variability of results due to individual differences, is still a topic of debate, the protective role of a diet rich in DF for overall health and gut integrity is well documented [143,144]. Conversely, the low DF intake characteristic of the WD has been associated with gut microbiota disruption, altered intestinal permeability, and subsequent low-grade inflammation [94,143]. Interestingly in our context, a study using a murine model has shown that long-term exposure to a WD leads to higher metabolic risks and a different gut microbiota composition in females compared to males. Aging, particularly the decrease in estrogen levels, along with diet could explain these results [145]. 

A diet rich in sugar, fat such as omega-6, SFA, CT, TFA, refined CHOs, and UPF food and poor in fibers, antioxidants, and omega-3 and 9 corresponds to the definition of the WD. The WD is associated with inflammation and may contribute to the development of mental health diseases [146].

## 4. Mood and Sleep Disturbances and Their Impacts on Inflammation

### 4.1. Psychological Stress and Gut Permeability

Stress management is a systemic and multifactorial physiological response which involves two endocrine systems differing in their pathways and reactivity: the sympathetic–adrenal–medullary (SAM) system and the hypothalamic–pituitary–adrenal (HPA) axis. In response to the stressor, SAM first triggers the sympathetic ganglion that leads to the release of catecholamines (among others) which in turn regulate the cardiovascular, skeletal muscle, hepatic, and pulmonary systems, thus constituting the first-line reaction [125]. At the same time, but more slowly, HPA triggers the central production of Corticotrophin Releasing Hormone (CRH), then the production of Adreno Cortico Trophic Hormone (ACTH) from the pituitary gland into the bloodstream. ACTH activates the adrenal cortex that in turn releases cortisol (in humans). While both axes could interact with the gut, the HPA axis could disrupt the gut’s permeability [125].

In animal models, evidence has shown that psychological or social stressors lead to an increase in the gut permeability in a CRH- and mast cell-dependent manner [147,148,149]. Vanuystel et al. have shown in healthy human subjects that psychological stress also increases small intestinal permeability [150]. Moreover, they have shown that CRH injection could mimic the effect of stressors on the gut barrier and this effect could be counteracted by the stabilization of the mast cell- as well as CRH-antagonist receptors [147,150]. The activation of mast cells leads to the release of pro-inflammatory cytokines [151]. In humans, a threshold of stressors could be necessary to lead to the disruption of gut permeability [125]. Another hypothesis is reliant on the nutrients needed in a stress context. Stress leads to an increase in the availability of nutrients, water, and electrolytes (including sodium) that can help the organism to cope with the presumed “danger”. Due to the humoral (e.g., catecholamine, cortisol) and nervous connection (adrenergic sympathetic nerve fibers) between the gut and brain, both axes (SAM and HPA) could contribute to increasing the paracellular permeability in elevating the diffusion of water and ions needed. Therefore, this could also allow the transfer of endotoxins that could in turn trigger low-grade inflammation [152,153]. Interestingly, in animal models, blockage of the sympathetic nervous system leads to a decrease in the permeability induced by endotoxins [154]. Further studies in humans need to be conducted due to the heterogeneity of markers and psychological stressors used, but evidence still shows that stress could also be involved in gut barrier disruption [150,152,153].

### 4.2. Sleep and Inflammation

Scientific research has established a strong connection between sleep disturbances and low-grade systemic inflammation, mediated by a range of interconnected physiological pathways. These include immune dysregulation, hormonal imbalances, and metabolic alterations [155]. Chronic sleep disturbances are linked to elevated levels of inflammatory markers such as CRP, interleukin-6 (IL-6), and tumor necrosis factor-alpha (TNF-α) [156,157], as well as increased stress hormones such as cortisol [158]. The mechanisms involved may operate through activation of the hypothalamic–pituitary–adrenal (HPA) axis, increasing cortisol levels, which normally suppresses inflammatory responses. However, prolonged activation leads to glucocorticoid resistance, allowing inflammatory genes (like IL-1β, IL-6, and TNF) to be expressed, while still suppressing antiviral genes (e.g., IFNα, IFNβ). At the same time, sympathetic nervous system (SNS) activation releases noradrenaline and adrenaline, which act on β2-adrenergic receptors in immune cells to further enhance inflammatory gene expression and suppress antiviral responses [155]. This shift toward inflammation may be mediated by pathways involving NF-κB, the NLRP3 inflammasome, and caspase-1, which can impair glucocorticoid receptor function. In parallel, sleep disturbances could negatively impact the gut microbiome, increasing gut permeability and systemic inflammation [159,160]. Notably, women may be more susceptible than men to sleep-related inflammatory responses, possibly due to sex-specific immune and hormonal differences [161,162].

**Figure 1:**
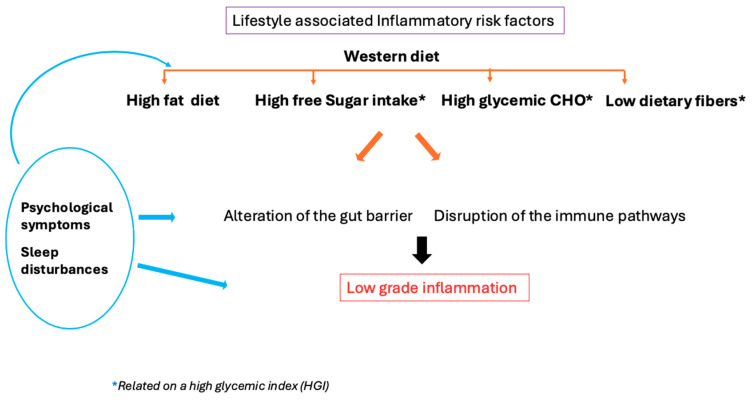
Psychological symptoms (mood disorder, stress) and sleep disturbance could enhance peripheral inflammation directly or indirectly through dietary patterns and gut integrity alteration.

Thus, perimenopause could create a vicious circle in which the same dietary pattern due to metabolic slowdown increases overweight. At the same time, mood disturbances and sleep deprivation could foster the consumption of HGI foods that could reinforce this deleterious context (Figure 1).

## 5. Inflammation, a Risk Factor for MDD and AD

Dantzer et al. first described sickness behavior theory (SB) as an overlap between the symptoms enduring in an infectious disease and those of depression such as depressive mood, anxiety, anhedonia (lack of pleasure), and sleep disturbance [163]. During an infectious episode, cytokines could cross the blood–brain barrier through more permissive pathways. Likewise, visceral inflammation or that due to a disrupted intestinal barrier could be sensed by the nerve’s afferent fibers. Both signals could trigger in the central nervous system (CNS) fever, pain, psychological symptoms (depressive mood, anhedonia, anxiety), sleep disturbance, and cognitive impairment [164,165]. SB has been seen as a positive evolution of adaptative immunity to ensure a withdrawn behavior which could foster recovery by saving energy. When the infection ends, so do the symptoms. In contrast, chronic low-grade inflammation due to lifestyle factors as aforementioned could continually enhance those symptoms and, in the case of preexisting depression, reinforce the psychiatric pathology. The interferon alpha treatment used for certain cancers or hepatitis C is clinical proof of the SB theory [166]. This treatment, used to boost a deficient immune system, leads to neurovegetative and somatic symptoms in all patients (e.g., sleep disturbance, pain, fever). In addition, around 40% of the patients suffer from delayed psychological symptoms such as depressive mood, anhedonia, and anxiety. Interestingly, those patients have been evaluated with higher preexisting psychological or genetic inheritance risk factors [166].

The idolamine/kynurenine pathway, which can be activated by inflammation, may also be involved. This pathway metabolizes tryptophan which is the precursor of serotonin. Serotonin is one of the most important neurotransmitters involved in mood homeostasis. Therefore, a depletion of tryptophan could lead to a depletion of serotonin and thus constitute a risk factor for depressive mood [165,167]. Finally, the Selective Serotonin Reuptake Inhibitor (SSRI), which is the most often prescribed drug against depression, has been shown less effective than other drugs such as tricyclic or SSRI–bupropion in very low-grade inflammation [168,169]. Thus, low-grade inflammation could reduce the effectiveness of the treatment depending on the level of the inflammatory state. More studies are needed to confirm this, but SSRIs prescribed in the case of a momentary depressive mood such as during menopause (or perimenopause) could be rather ineffective. In the case of preexisting depression, inflammation is associated with a more serious diagnosis: increase in suicide attempts, relapses, symptom enhancement, treatment resistance, and a higher prevalence of cardiometabolic comorbidities [170,171,172].

Chronic low-grade inflammation is also recognized as a hallmark of AD and a significant contributor to its progression, particularly in the acceleration of cognitive decline [173]. In individuals with AD, peripheral inflammation is marked by elevated levels of circulating pro-inflammatory cytokines, including IL-1β, IL-6, TNF-α, or high-sensitivity CRP, when compared to cognitively healthy individuals [174]. This systemic inflammatory state can compromise the integrity of the blood–brain barrier (BBB) by altering tight junction proteins, damaging vascular endothelial cells and degrading the endothelial glycocalyx [175]. As a result, immune cells, cytokines, and other pro-inflammatory mediators can more readily enter the brain, promoting a neuroinflammatory environment and facilitating the accumulation of amyloid-beta (Aβ) in the central nervous system [176]. Beyond diffusion through a weakened BBB, pro-inflammatory substances may also access the brain via circumventricular organs, or through active transport mechanisms [177]. In parallel, systemic inflammatory signals can be relayed to the brain via peripheral nerves, particularly the vagus nerve which serves as a primary bidirectional communication pathway between the peripheral immune system and the central nervous system [178]. Once within the brain, these pro-inflammatory signals activate microglia and astrocytes, shifting them into a reactive state. This chronic activation impairs their ability to clear pathological proteins and fosters a feed-forward inflammatory cycle, which accelerates the formation of extracellular amyloid plaques and intracellular neurofibrillary tangles—the pathological hallmarks of AD [179].

## 6. Conclusions

**Figure 2:**
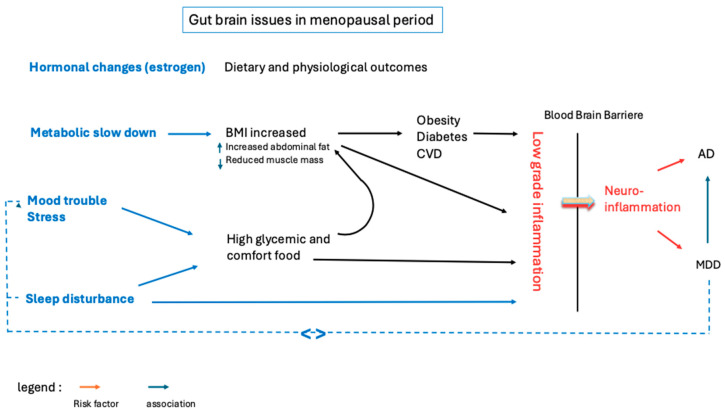
Hormonal changes can lead to mood and sleep disturbances, and a slowed metabolism, which may affect dietary intake and increase abdominal fat. Overweight, comfort food, a Western diet, or all of these could thereby promote low-grade inflammation (LGI). LGI could cross the blood–brain barrier and subsequently act as a risk factor for major depression and Alzheimer’s disease.

Menopause is a critical period in a woman’s life that extends far beyond reproductive aging, encompassing profound hormonal, metabolic, and neurobiological changes. The interconnected effects on mood, sleep, and brain health remain under-recognized. Emerging evidence underscores the role of chronic low-grade inflammation as a common thread linking menopausal symptoms with increased susceptibility to depression and neurodegenerative diseases such as Alzheimer’s. Nutritional behaviors, particularly the adoption or reinforcement of a Western dietary pattern in the context of sleep and mood disorders, may exacerbate this inflammatory state—contributing to metabolic dysregulation and altering the gut–brain axis. These combined factors may, over time, impair neuronal function and compromise mental well-being (Figure 2). Given the growing aging population and the underrepresentation of women in clinical research, personalized, integrative, and preventive strategies during menopause—including those targeting diet, sleep, and mental health—may offer a non-invasive path for preventing or delaying the onset of mood and neurodegenerative disorders.
